# Changing of the guards

**DOI:** 10.3402/ijch.v74.28917

**Published:** 2015-07-06

**Authors:** Kue Young, Rhonda Johnson

**Affiliations:** 1 School of Public Health, University of Alberta, Edmonton, Canada; 2 University of Alaska Anchorage, Anchorage, AK, USA

The *International Journal of Circumpolar Health* reached another milestone in June 2015 with a change of the chief editorship from Kue Young (University of Alberta, Canada) to Rhonda Johnson (University of Alaska Anchorage, United States).

Over the years, the journal has undergone several major changes. It might be helpful to our readers to highlight them:

- Establishment in 1973 as an organ of the Nordiska Samarbetskommitten för Arktisk Medicinsk Forskning (NoSAMF), reporting to and financed by the Nordic Council of Ministers (NCM).
- The dissolution of NoSAMF in 1996 and the transfer of the journal to joint ownership by the Nordic Society for Arctic Medicine (NSAM) and the International Union of Circumpolar Health (IUCH). Editorial and administrative functions were assumed by the Centre for Arctic Medicine at the University of Oulu.
- Changing of the name from *Arctic Medical Research* to *International Journal of Circumpolar Health* in 1997.
- Creation of the International Association of Circumpolar Health Publishers (IACHP), a consortium of several universities, research institutes and professional/academic organizations as the publisher of the journal in 2004.
- Merging of IACHP with the International Network for Circumpolar Health Research (INCHR) to form the Circumpolar Health Research Network (CHRN) in 2013, which became the publisher of the journal.
- The closing of the editorial office at the University of Oulu, the publication agreement between CHRN and Co-Action Publisher and the change to a completely on-line journal in 2012.
- The institution of publication fees in 2013.

As outgoing and incoming editors-in-chief, we firmly believe that *IJCH* serves an essential function in the dissemination and translation of scientific knowledge on all aspects of circumpolar health to our community of health researchers, providers, managers, policy makers and community leaders. The quality and impact of the journal continues to improve, thanks not only to the increasing range of scientific endeavour in the arctic but also to the countless hours of volunteer effort by our growing ranks of peer reviewers willing to share their expert critique of manuscripts upon request. If you are not yet on our reviewer list and would like to be, please contact Rhonda Johnson. And if you are engaged in meaningful and interesting work related to circumpolar health, please consider sharing your experiences and lessons learned with our readers through submission of manuscripts. Check out the “For Authors” tab, and consider joining the on-line discussions via Facebook, Twitter and LinkedIn. We welcome your contributions to the continuing success of our journal.

Kue Young School of Public Health University of Alberta Edmonton, Canada Rhonda Johnson University of Alaska Anchorage Anchorage, AK, USA

